# Supplementary material for: The cytoplasmic synthesis and coupled membrane translocation of eukaryotic polyphosphate by signal-activated VTC complex
Source: Nat Commun. 2023 Feb 9;14:718. doi: 10.1038/s41467-023-36466-4 (PMC9911596; doi:10.1038/s41467-023-36466-4)
Supplement: Supplementary file 1 — Supplementary Information [file 41467_2023_36466_MOESM1_ESM.pdf]

# Supplementary Information for

# The cytoplasmic synthesis and coupled membrane translocation of eukaryotic polyphosphate by signal-activated VTC complex

Zeyuan Guan[#], Juan Chen[#], Ruiwen Liu[#], Yanke Chen[#], Qiong Xing[#], Zhangmeng Du, Meng Cheng, Jianjian Hu, Wenhui Zhang, Wencong Mei[1], Beijing Wan[1], Qiang Wang, Jie Zhang, Peng Cheng, Huanyu Cai, Jianbo Cao, Delin Zhang, Junjie Yan, Ping Yin, Michael Hothorn, Zhu Liu[*]

[#]These authors contributed equally to this work.

[*]Corresponding author: liuzhu@hzau.edu.cn (Z. L.).

**This PDF file includes:**

Supplementary Figs. 1-15

Supplementary Tables 1-3

References

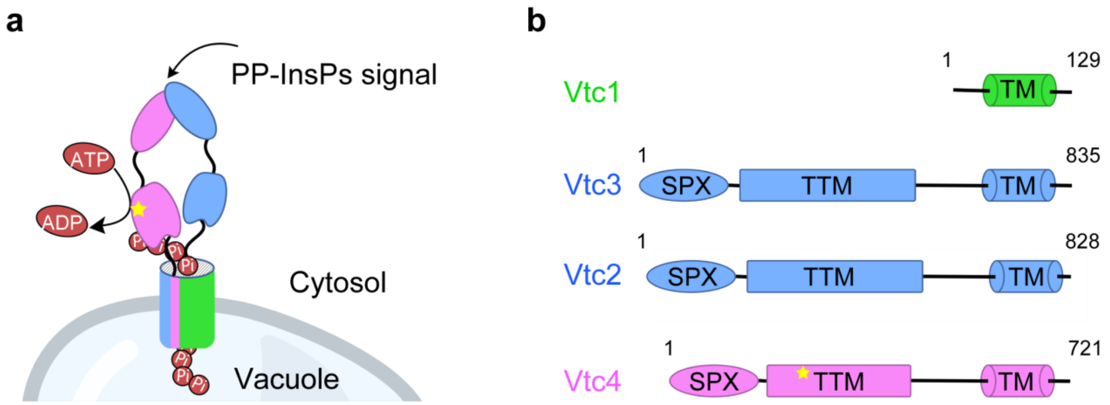


**Supplementary Figure 1.** (**a**) Schematic working model and (**b**) domain architectures of Vtc subunits. TM, trans-membrane domain; SPX, SYG1/Pho81/XPR1 domain; TTM, triphosphate tunnel metalloenzyme domain. A yellow star indicates the catalytic polymerase domain for polyP synthesis.

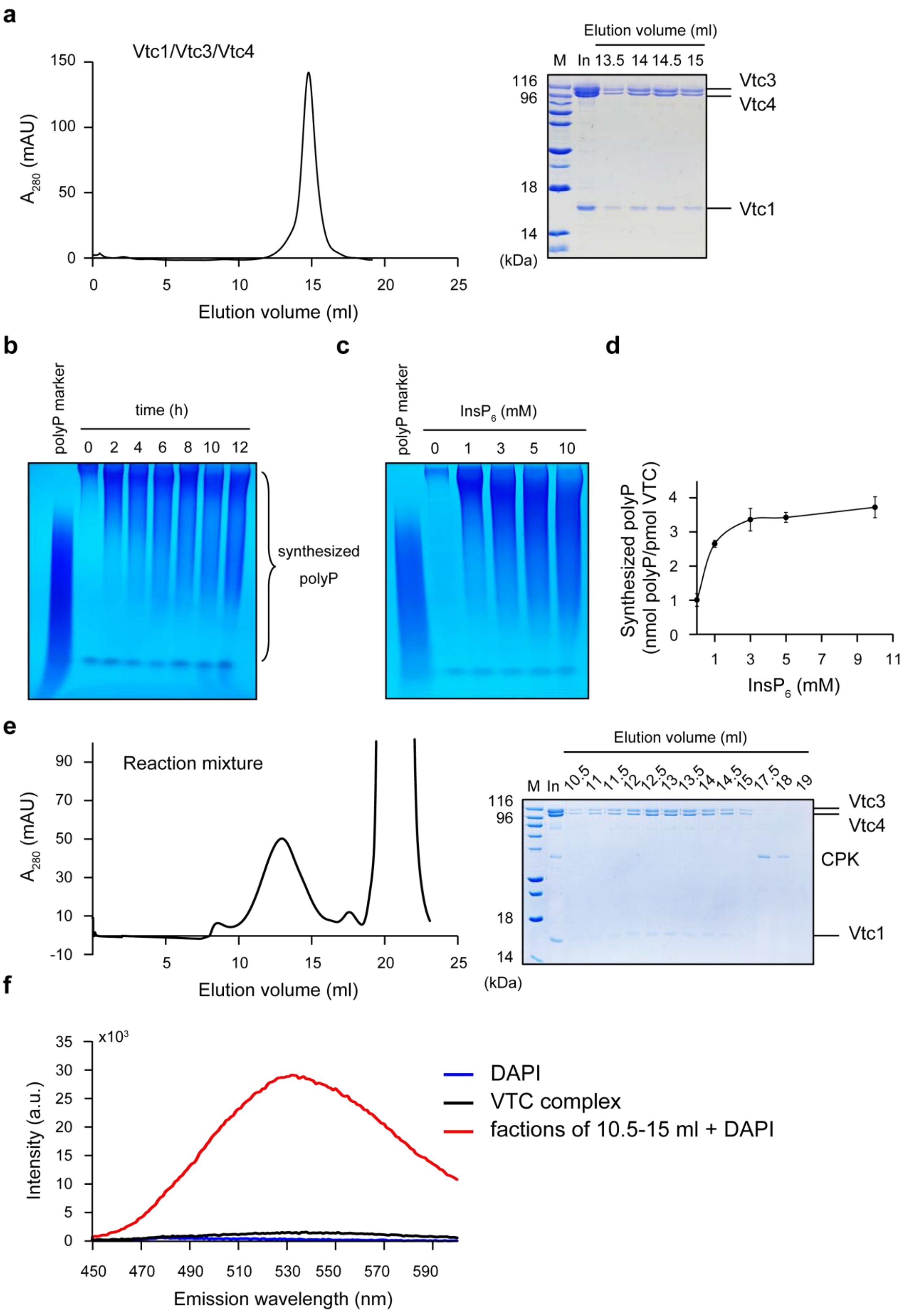


**Supplementary Figure 2. Purification of the VTC complex and polyP activity assay.** (**a**) Representative size-exclusion chromatography (SEC) purification of the Vtc1/Vtc3/Vtc4 complex in the absence of $InsP_6$. In, input sample before SEC step. Source data are provided as a Source Data file. The experiments were repeated more than five times independently with similar results. (**b**) TBE-PAGE visualization of polyP synthesized by the Vtc1/Vtc3/Vtc4 complex. The reactions

were performed with required times before stopped. 5 μM protein complex and 10 mM $InsP_6$ were used for each reaction. A commercial polyP with an average chain length of 60 residues (polyP60) was used as the marker. Source data are provided as a Source Data file. The experiments were repeated three times independently with similar results. (**c**) TBE-PAGE visualization of polyP synthesized by Vtc1/Vtc3/Vtc4 complex. The reactions were performed at required $InsP_6$ concentrations. 5 μM protein complex were used and reactions were performed for 4 hours. Source data are provided as a Source Data file. The experiments were repeated three times independently with similar results. (**d**) Quantification of the produced polyP from (**c**) using DAPI-based measurement[1]. PolyP60 was used for assay calibration. Data from three measurements were averaged, and the error indicates SD. Source data are provided as a Source Data file. (**e**) SEC analysis of the reaction mixture. 5 μM protein complex and 10 mM $InsP_6$ were used and the reaction of polyP synthesis was performed for 2 hours. Then the reaction mixture was subjected onto SEC. Fractions of eluent were visualized on SDS-PAGE gel. In, input reaction mixture before SEC step. CPK, creatine phosphokinase used in the reaction. Source data are provided as a Source Data file. The experiments were repeated two times independently with similar results. (**f**) DAPI detecting the retained polyP in the collected SEC fractions of the reaction mixture. Fractions of VTC at 10.5-15 ml elution volume of (**e**) were collected and concentrated for the DAPI detection. 0.2 μM concentrated protein fractions and 10 μM DAPI were used for the fluorescent detection. As controls, 0.2 μM purified VTC complex and 10 μM DAPI were used, respectively. The experiments were repeated two times independently with similar results.

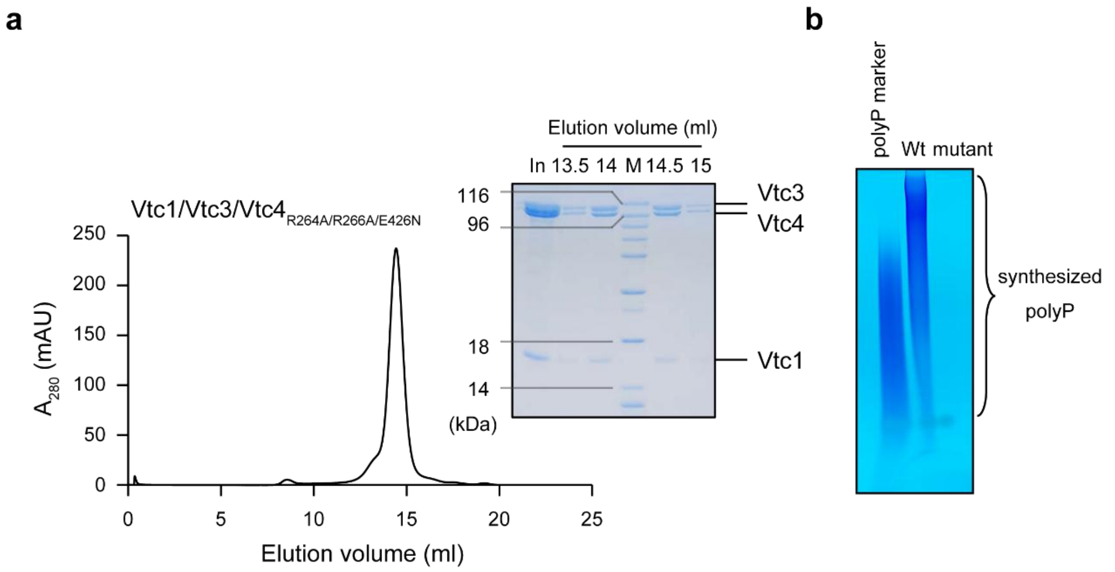


**Supplementary Figure 3. Purification of the VTC complex for cryo-EM analysis.** (**a**) Representative SEC purification of the Vtc1/Vtc3/Vtc4 complex carrying three point mutations on Vtc4 ($Vtc1/Vtc3/Vtc4_{R264A/R266A/E426N}$) in the presence of 1 mM $InsP_6$. In, input sample before SEC step. Fractions of $Vtc1/Vtc3/Vtc4_{R264A/R266A/E426N}$ at 14 and 14.5 ml elution volume were pooled for cryo-EM analysis. Source data are provided as a Source Data file. The experiments were repeated more than five times independently with similar results. (**b**) TBE-PAGE visualization of polyP synthesized by Vtc1/Vtc3/Vtc4 (Wt) and $Vtc1/Vtc3/Vtc4_{R264A/R266A/E426N}$

(mutant) complex. 5 µM protein complex and 10 mM $InsP_6$ were used, and reactions were performed for 4 hours. Source data are provided as a Source Data file. The experiments were repeated three times independently with similar results. Blocking VTC activity via three amino-acid substitutions of known catalytic residues in $TTM^{VTC4}$ (R264A/R266A/E426N)[2], allowed us to collect high-quality cryo-EM micrographs for single particle analysis.

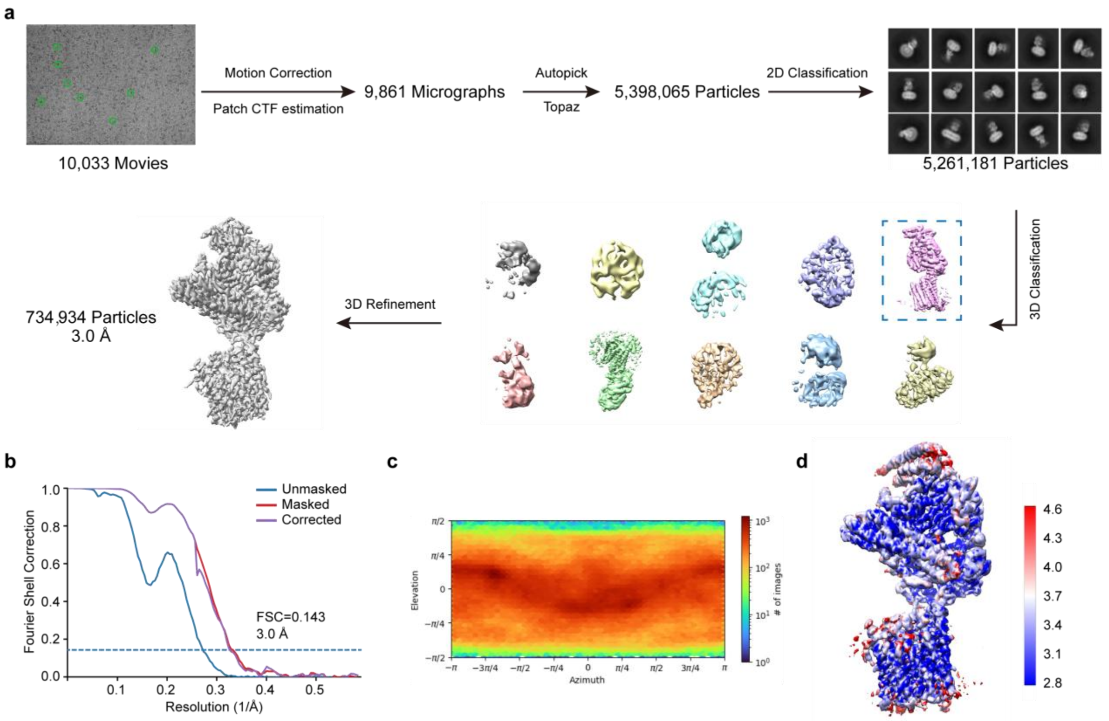


**Supplementary Figure 4. Cryo-EM structural analysis of the VTC complex.** (**a**) Flow chart for EM data processing. 3D refinement generated a 3.0 Å map of Vtc1/Vtc3/$Vtc4_{R264A/R266A/E426N}$ in complex with $InsP_6$. Typical particles of the complex are marked by green circles in the representative electron micrograph. Details can be found in Methods. (**b**) Gold-standard Fourier shell correlation (FSC) curve for the VTC map refinement. Average resolution of this map is estimated to be 3.0 Å. (**c**) Angular distribution of the particles used for reconstruction. (**d**) Sharpened map of the complex, colored according to local resolution.

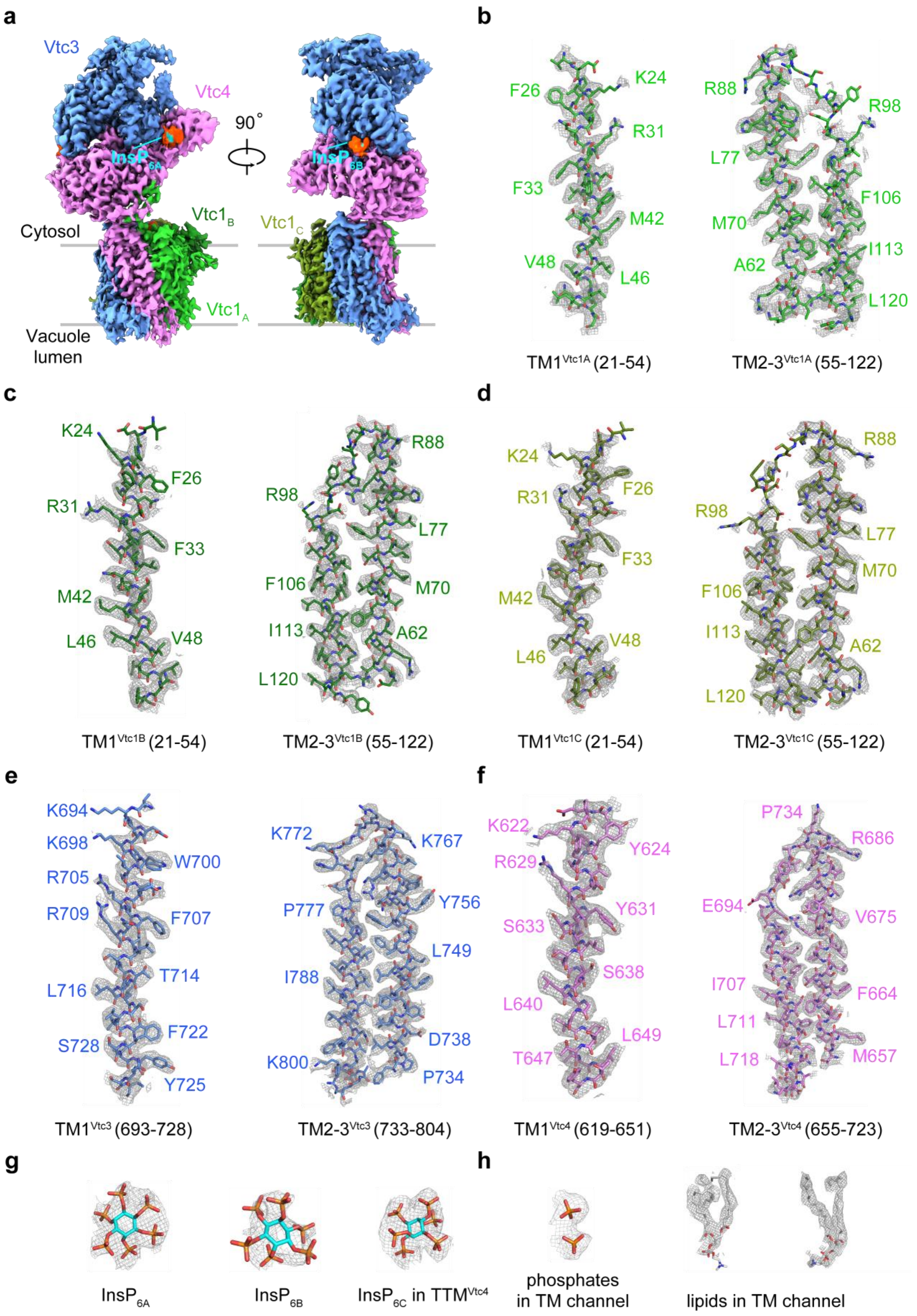


**Supplementary Figure 5. Representative EM densities.** (**a**) The EM density map of the Vtc1/Vtc3/Vtc4$_{R264A/R266A/E426N}$ in complex with InsP$_6$. (**b-f**) EM density maps for the indicated trans-membrane helices in the TM region of the complex. (**g**) and (**h**), EM density maps for the indicated molecules observed in the complex. All the densities were contoured at 5.5 σ and visualized using PyMol 2.4.1.

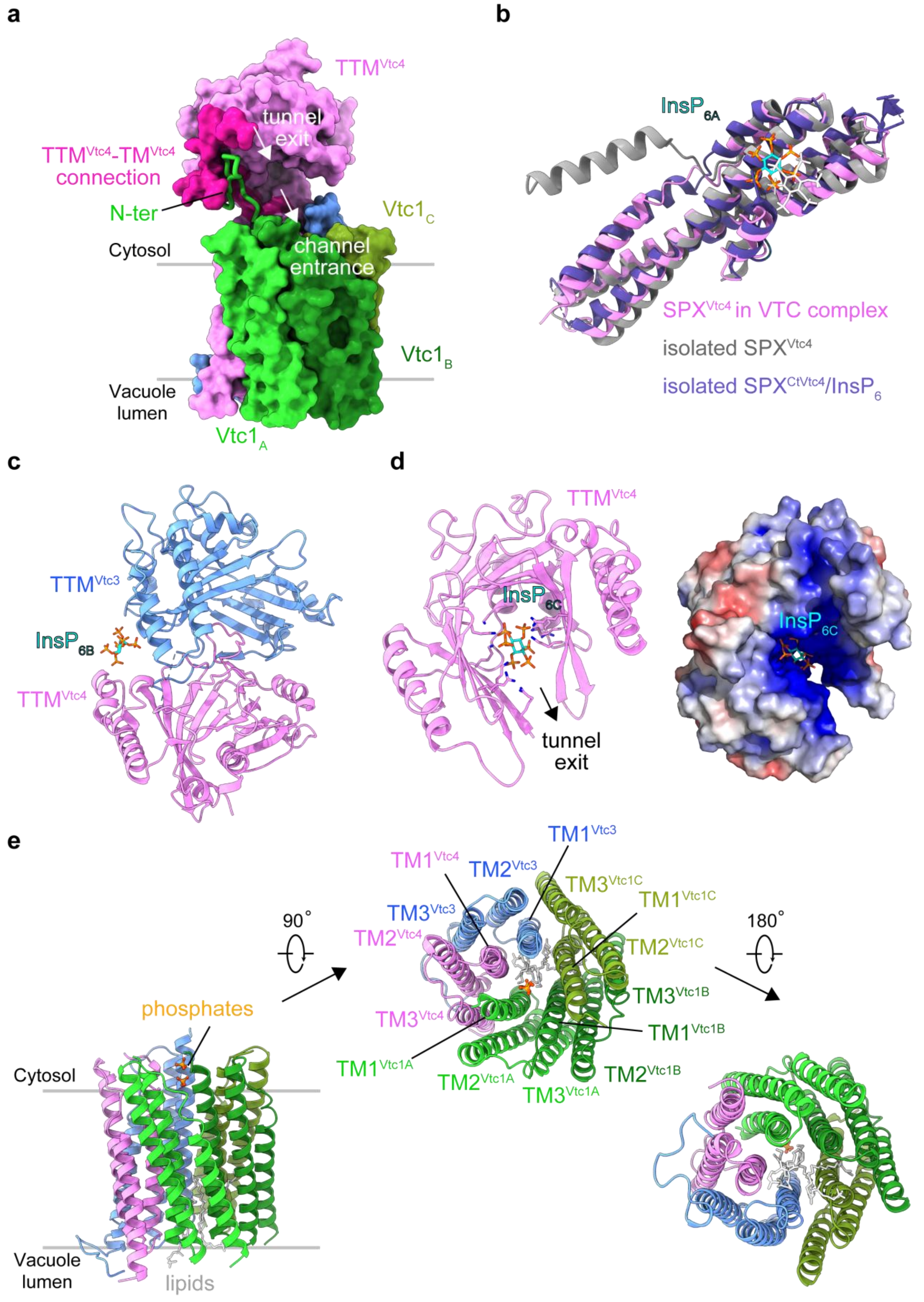


**Supplementary Figure 6. Structural segments of the InsP$_6$-activated VTC complex.** (**a**) Orientation between the catalytic TTM$^{Vtc4}$ domain and the assembled trans-membrane channel. The connection between TTM$^{Vtc4}$ and TM$^{Vtc4}$ (residues 472-618) is represented in magenta surface. The N-terminus of Vtc1$_A$ protomer (residues 1-21) is shown in cartoon representation. (**b**) Structure comparison between InsP$_6$-bound SPX$^{Vtc4}$ in our entire yeast VTC complex and

previously reported isolated SPX fragments. The structure of isolated yeast $SPX^{Vtc4}$ fragment (5IIT.PDB)[3] and isolated *Chaetomium thermophilum* $SPX^{CtVtc4}$ fragment in complex with $InsP_6$ (5IJP.PDB)[3] are colored in gray and purple cartoon representations, respectively. $InsP_6$ of 5IJP.PDB is shown in white stick representation. (**c**) The second $InsP_6$ molecule ($InsP_{6B}$) binds simultaneously to $TTM^{Vtc3}$ and $TTM^{Vtc4}$. (**d**) The third $InsP_6$ molecule ($InsP_{6C}$) appears in the positively charged tunnel of $TTM^{Vtc4}$. Positive residues in inner wall of the tunnel are shown as stick in the cartoon representation of $TTM^{Vtc4}$. Electrostatic surface is represented in the same perspective, colored in terms of electrostatic potential and displayed in a scale from red (−5 kT/e) to blue (+5 kT/e). (**e**) Molecules of two phosphates and two lipids occupy in the cytoplasmic side and luminal side of trans-membrane channel, respectively. Phosphates and lipids are shown in stick representations.

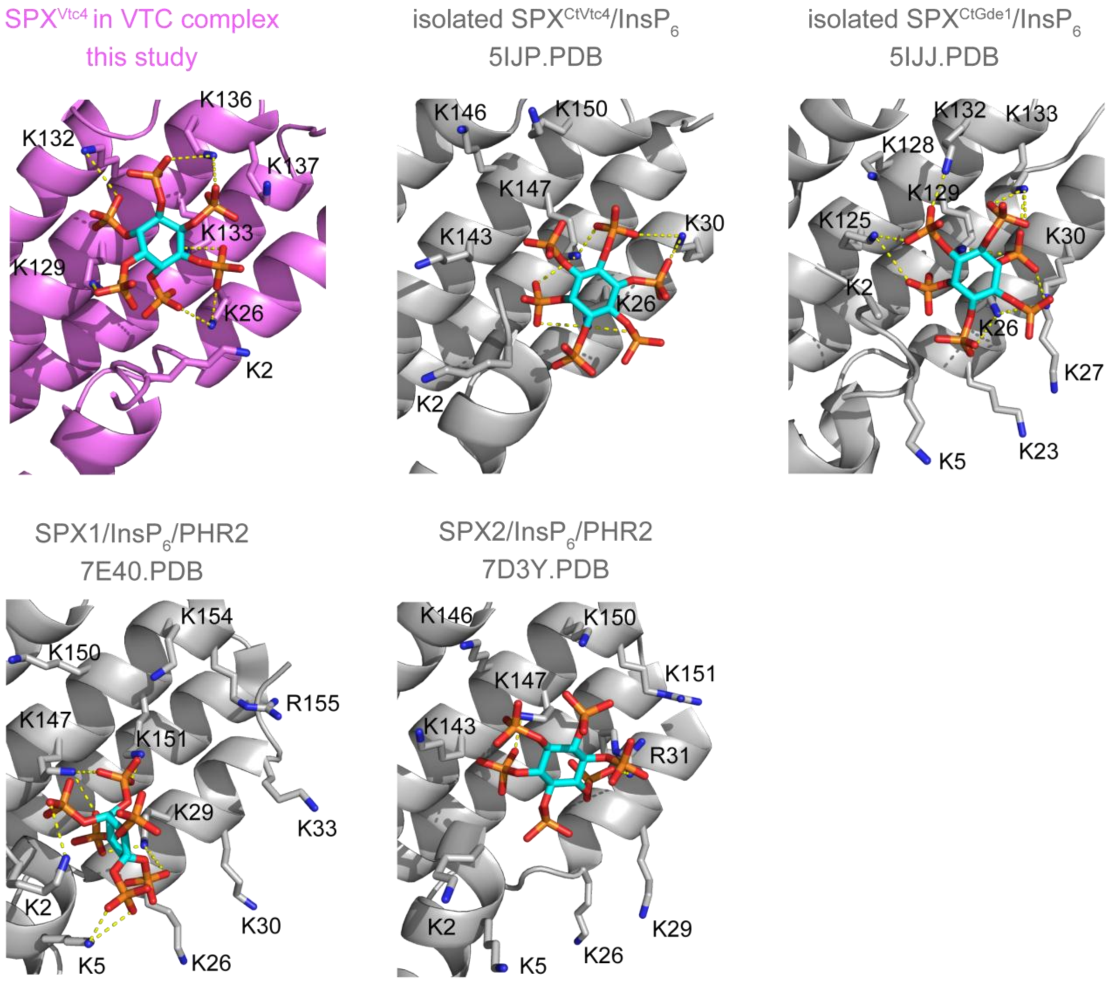


**Supplementary Figure 7. Coordination of $InsP_6$ bound to different SPX domain-containing proteins.** All SPX domains (depicted as cartoon diagrams) are shown in the same orientations. Other proteins in the complex structures have been omitted for clarity. Positively charged residues constituting the basic surface patch in SPX proteins are shown in stick representation. Yellow dashed lines indicate the coordination of $InsP_6$.

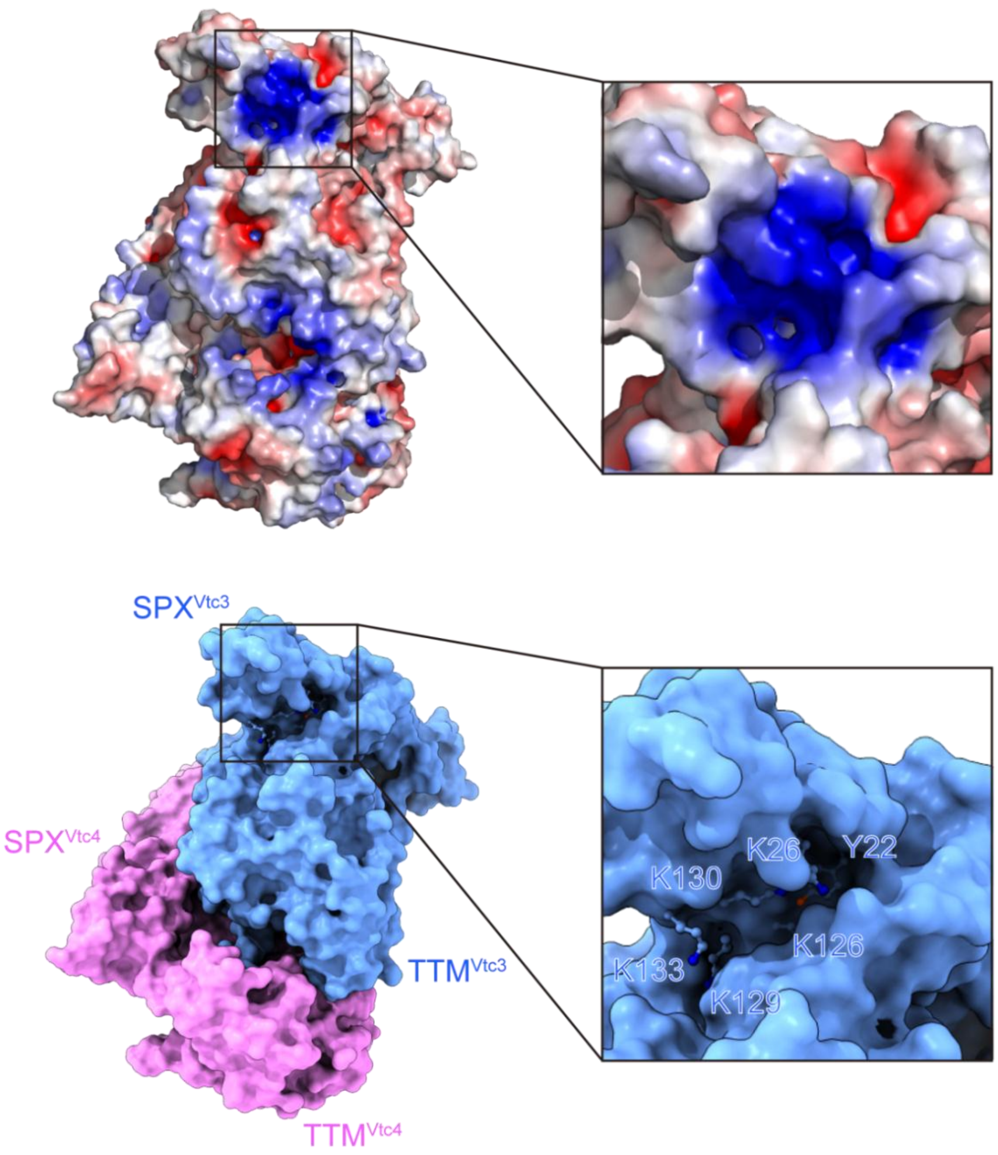


**Supplementary Figure 8. Electrostatic surface (upper panel) and molecular surface (lower panel) representation of SPX and TTM domains in the $InsP_6$-activated VTC complex.** They are displayed in the same perspective. Electrostatic potential is calculated with the APBS plugin in PyMOL, colored and displayed in a scale from red (−5 kT/e) to blue (+5 kT/e). $SPX^{Vtc3}$ PBC (Y22, K26, K130) and KSC (K126, K129, K133) residues are shown in stick representation.

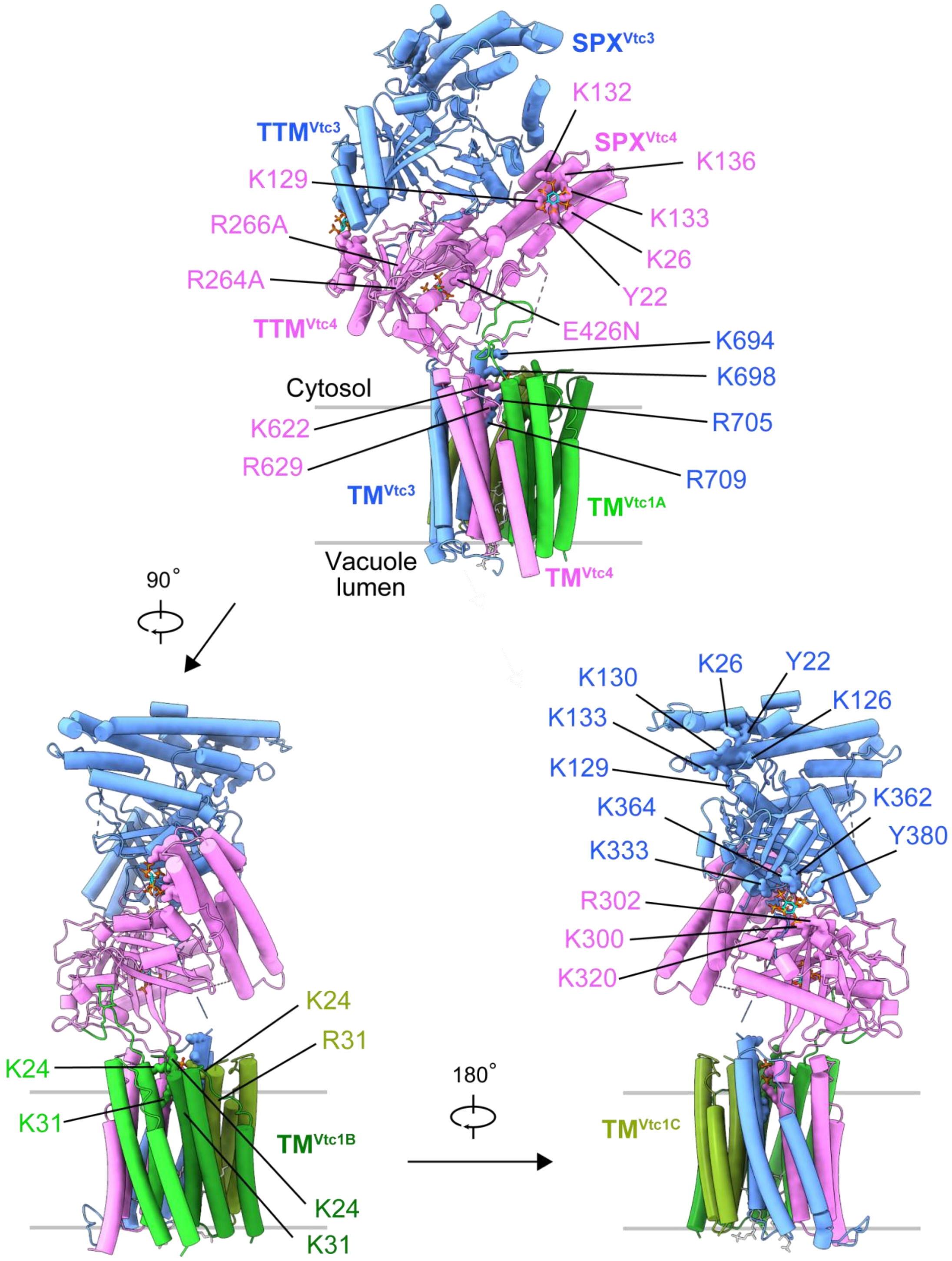


**Supplementary Figure 9. Sites of point mutations used in this study.** The VTC complex structure is presented in same scheme as Fig. 1 in the main text. Mutations of subunit Vtc4 R264A/R266A/E426N were used for structure determination, and others were used for function studies.

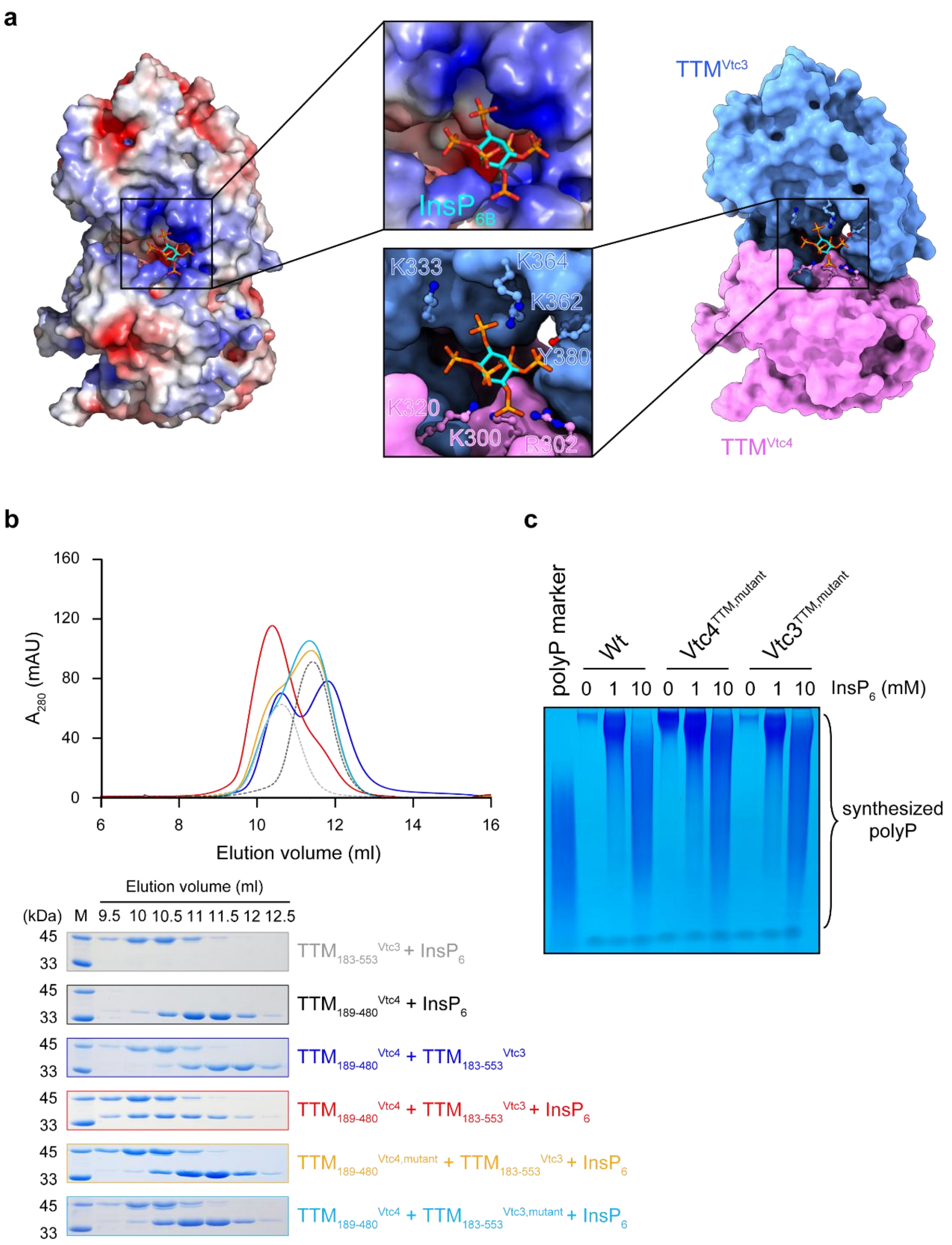


**Supplementary Figure 10. An $InsP_6$ molecule is coordinated between $TTM^{Vtc4}$ and $TTM^{Vtc3}$ in the $InsP_6$-activated VTC complex.** (**a**) $InsP_{6B}$ binds to a positively charged cleft that is formed by $TTM^{Vtc4}$ and $TTM^{Vtc3}$. The Electrostatic surface (left) and molecular surface (right) representation of $TTM^{Vtc3}$ and $TTM^{Vtc4}$ domains are displayed in the same perspective. Electrostatic potential is calculated with the APBS plugin in PyMOL, colored and displayed in a scale from red (−5 kT/e) to blue (+5 kT/e). Residues for $InsP_6$ coordination are shown in stick representation. (**b**) $InsP_{6B}$ promotes $TTM^{Vtc4}$-$TTM^{Vtc3}$ association. The isolated $TTM_{189\text{-}480}{}^{Vtc4}$ and $TTM_{183\text{-}553}{}^{Vtc3}$ contain residues 189-480 and residues 183-553, respectively. $TTM_{189\text{-}480}{}^{Vtc4,mutant}$ and $TTM_{183\text{-}553}{}^{Vtc3,mutant}$ carry K300A/R302A/K320A and K333A/K362A/K364A/Y380F point

mutations, respectively. (*Top*) Gel filtration profiles of $TTM_{183\text{-}553}^{Vtc3}$, $TTM_{189\text{-}480}^{Vtc4}$, $TTM_{189\text{-}480}^{Vtc4}$ and $TTM_{183\text{-}553}^{Vtc3}$ mixture, $TTM_{189\text{-}480}^{Vtc4,mutant}$ and $TTM_{183\text{-}553}^{Vtc3}$ mixture, $TTM_{189\text{-}480}^{Vtc4}$ and $TTM_{183\text{-}553}^{Vtc3,mutant}$ mixture in the presence of 1 mM $InsP_6$, and $TTM_{189\text{-}480}^{Vtc4}$ and $TTM_{183\text{-}553}^{Vtc3}$ mixture in the absence of $InsP_6$ were colored in gray, black, red, yellow, cyan and blue, respectively. (*Bottom*) Coomassie-blue stained SDS-PAGE gels of each peak fractions. About 25 μM protein or 50 μM mixture (25 μM for each) were used for the SEC assay. Source data are provided as a Source Data file. The experiments were repeated two times independently with similar results. (**c**) Assay of VTC-catalyzed polyP synthesis. Cleft mutations in either $TTM^{Vtc4}$ ($Vtc4^{TTM,mutant}$, $Vtc1/Vtc3/Vtc4_{K300A/R302A/K320A}$) or $TTM^{Vtc3}$ ($Vtc3^{TTM,mutant}$, $Vtc1/Vtc3_{K333A/K362A/K364A/Y380F}/Vtc4$) had little impact on $InsP_6$-stimulated polyP synthesis by VTC complex. 5 μM protein complex were used and reactions were performed at required $InsP_6$ concentrations for 4 hours. Source data are provided as a Source Data file. The experiments were repeated two times independently with similar results.

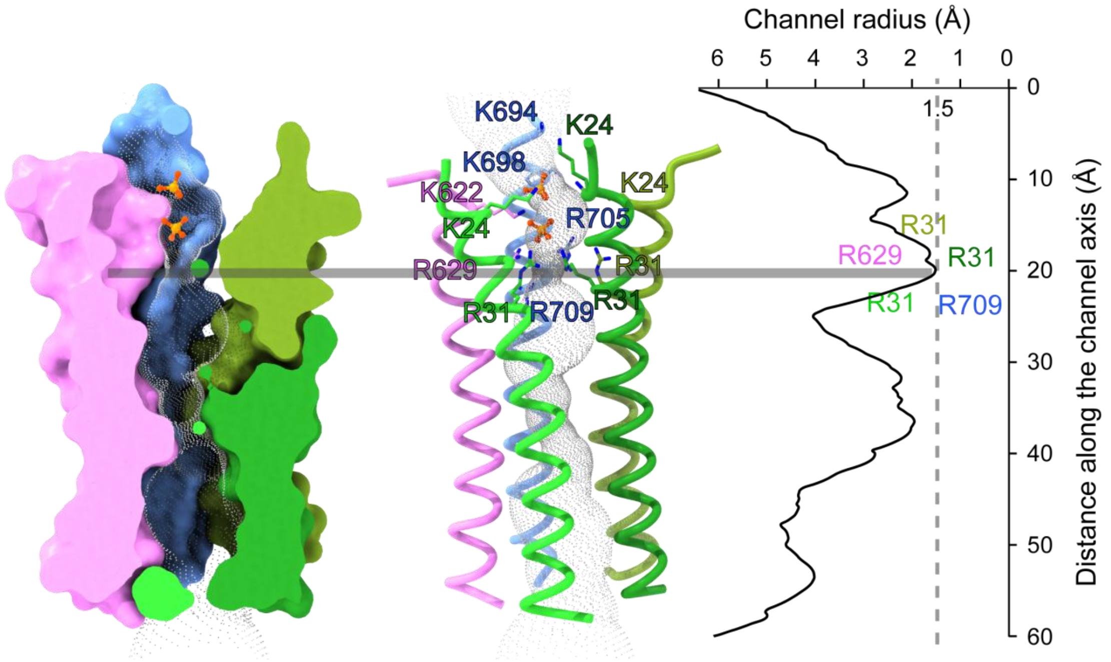


**Supplementary Figure 11. Channel path analysis.** Surface representation with half-cut view (left) and cartoon representation (middle) of the TM1s-assembled trans-membrane channel. Basic residues of the five TM1s in the interior wall and the occupied phosphates are shown in stick representations. Channel path (gray dots representation) and channel radius (right) was calculated using HOLE[4] for the VTC complex structure, without the inside phosphates and lipids. The dashed line indicates the narrowest position of the channel, that is surrounded by R31 of $TM1^{Vtc1}$, R709 of $TM1^{Vtc3}$, and R629 of $TM1^{Vtc4}$, with a radius of 1.5 Å. The VTC complex structure is colored in same scheme as Fig. 4b in the main text.

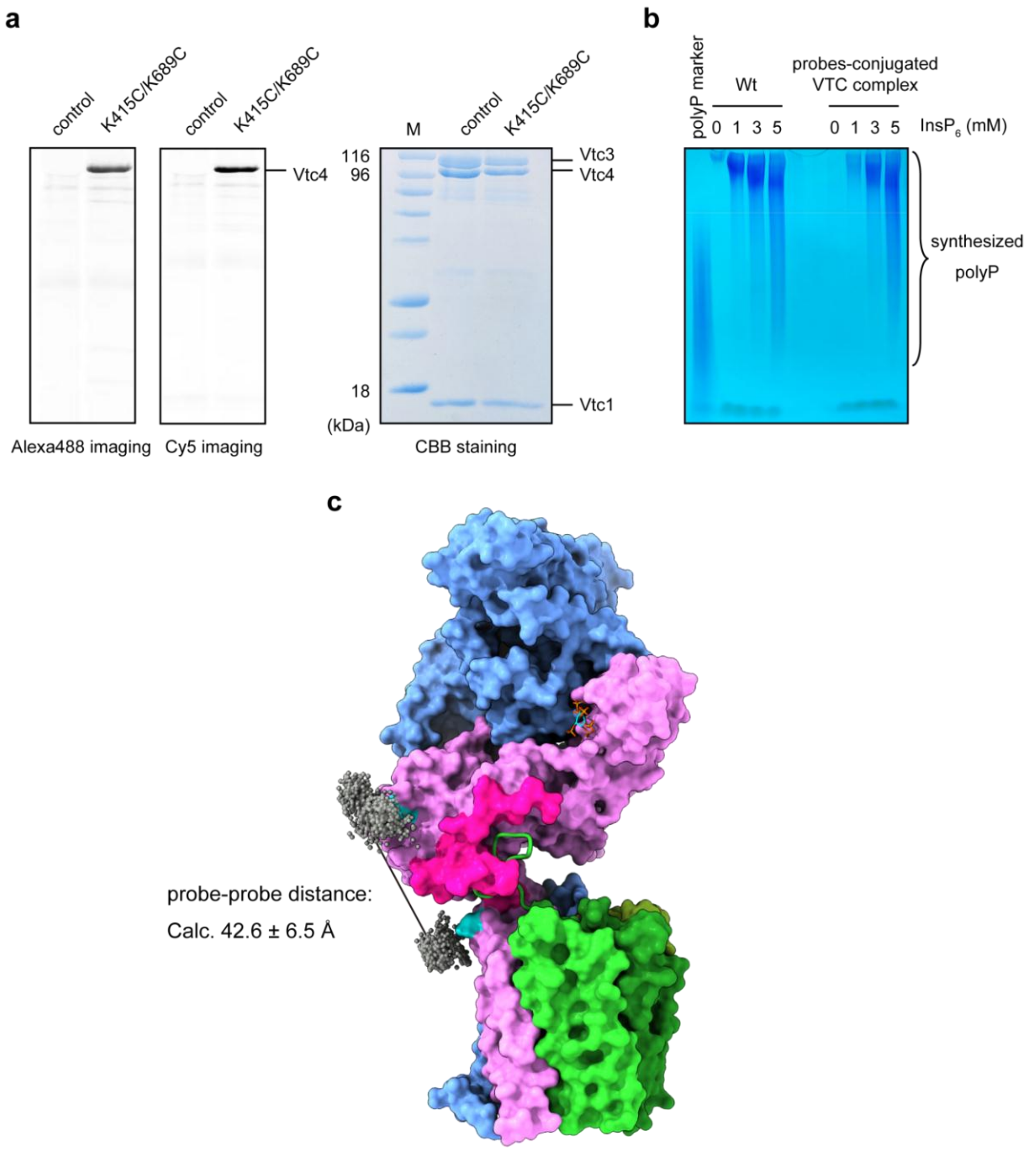


**Supplementary Figure 12. Sample used for smFRET analysis.** (**a**) Specifically labeling of the VTC complex. Control, all intrinsic cysteine in the Vtc1/Vtc3/Vtc4 complex were substituted; K415C/K689C, substituting all intrinsic cysteine in the VTC complex and further generating additional cysteine to K415 and K689 in the Vtc4 subunit. The images of Alexa488 and Cy5 on SDS-PAGE gel were obtained using Typhoon, and the gel was further stained using coomassie brilliant blue (CBB). Source data are provided as a Source Data file. The experiments were repeated three times independently with similar results. (**b**) Assay of VTC-catalyzed polyP synthesis. Wt, the Vtc1/Vtc3/Vtc4 wild-type complex; probes-conjugated VTC complex, the sample used for smFRET analysis. 5 μM protein complex were used and reactions were performed at required $InsP_6$ concentrations for 3 hours. Source data are provided as a Source Data file. The experiments were repeated three times independently with similar results. (**c**) Calculated average distance between conjugated probes. The accessible volume of conjugated probes on the structure were sampled using Xplor-NIH[5] (See Methods for details). The geometric centers of the two chromophores were represented with gray spheres, and the average distance between them is 42.6 ±6.5 Å. The VTC complex structure is colored in same scheme as Fig. 5c in the main text.

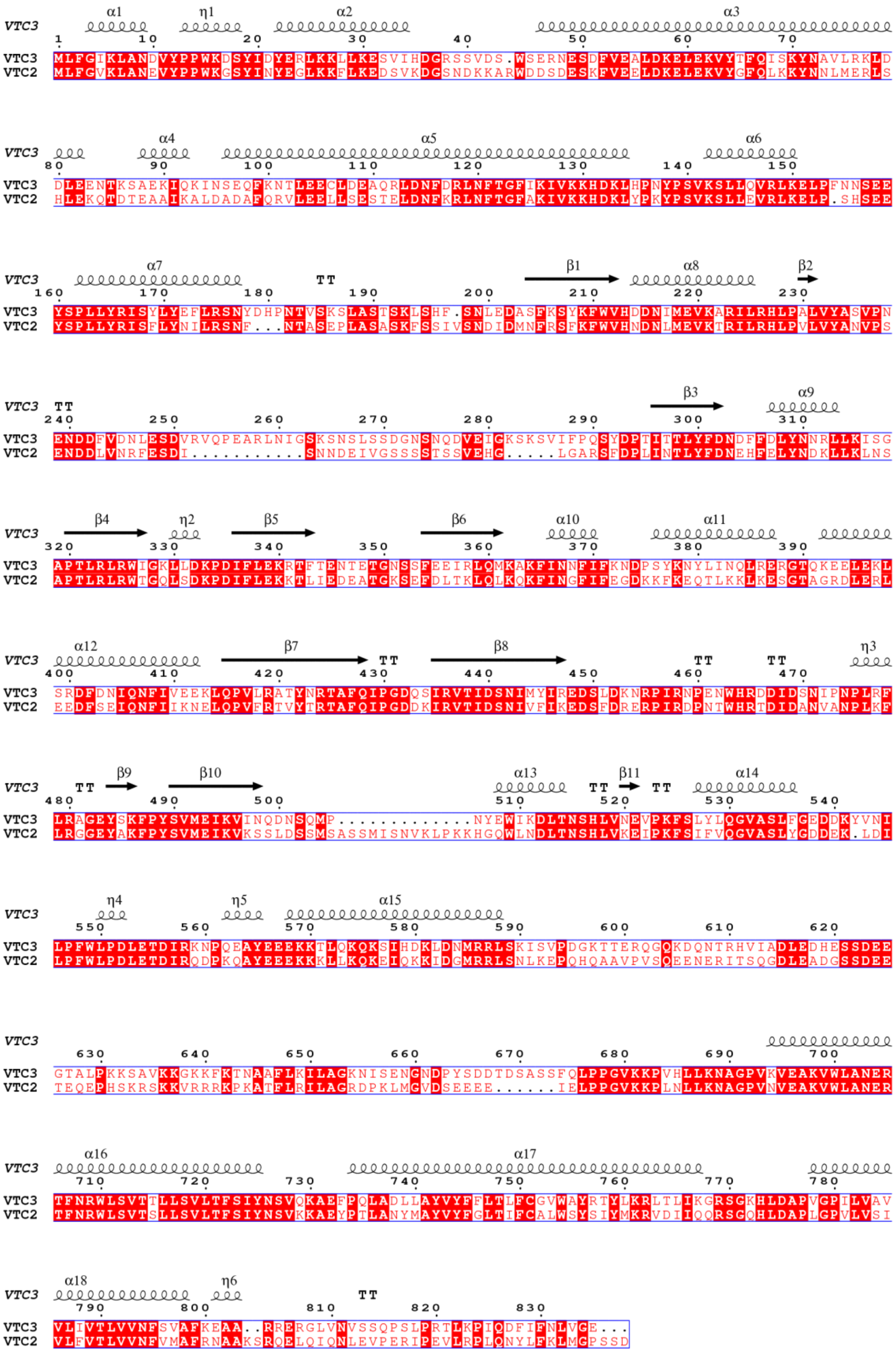

**Supplementary Figure 13. Sequence alignment.** The sequence of yeast Vtc3 subunit is aligned with Vtc2. Structure-based alignment was performed by ESPript (3.0). The sequence identity is indicated by white letters against a red background, and the sequence of a similarity over 90% is indicated by red letters. The secondary elements of Vtc3 are labeled at the top of the alignment.

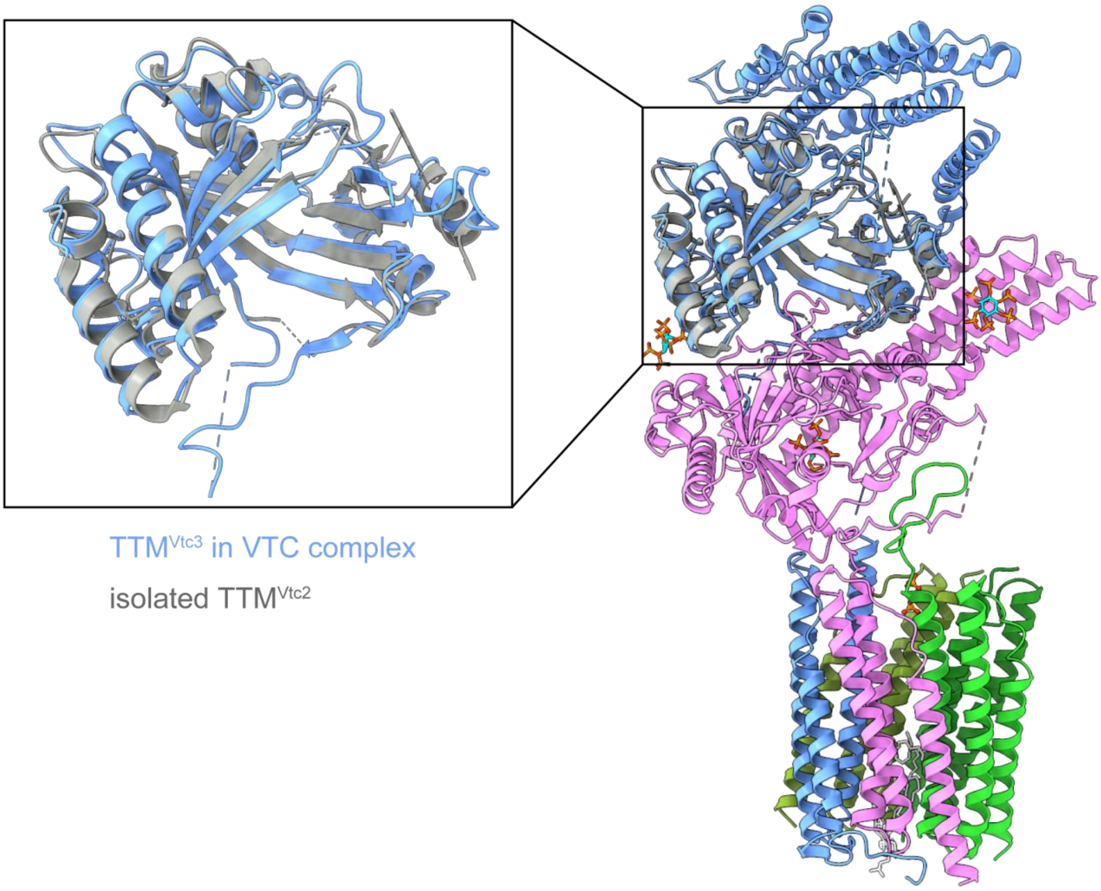


**Supplementary Figure 14. Structure comparison.** The structure of isolated TTM$^{Vtc2}$ fragment[2] (3G3O.PDB) is superposed to the TTM$^{Vtc3}$ domain in the InsP$_6$-activated Vtc1/Vtc3/Vtc4 complex structure, with an overall RMSD of 1.03 Å.

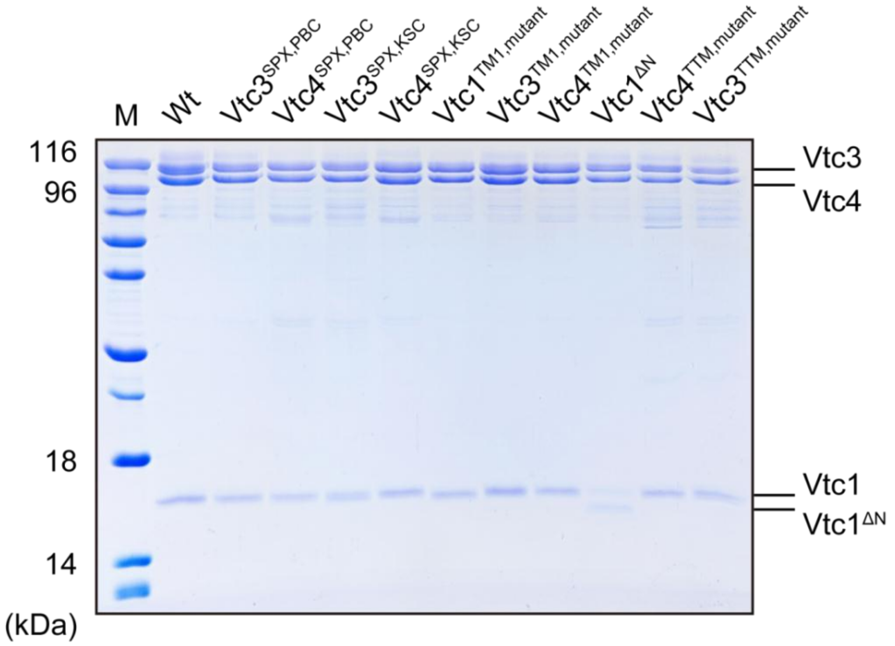


**Supplementary Figure 15. Samples used for the assay of VTC-catalyzed polyP synthesis in this study.** Wt, Vtc1/Vtc3/Vtc4; $Vtc3^{SPX,PBC}$, $Vtc1/Vtc3_{Y22F/K26A/K130A}/Vtc4$; $Vtc4^{SPX,PBC}$, $Vtc1/Vtc3/Vtc4_{Y22F/K26A/K133A}$; $Vtc3^{SPX,KSC}$, $Vtc1/Vtc3_{K126A/K129A/K133A}Vtc4$; $Vtc4^{SPX,KSC}$ $Vtc1/Vtc3/Vtc4_{K129A/K132A/K136A}$; $Vtc1^{TM1,mutant}$, $Vtc1_{K24A/R31A}/Vtc3/Vtc4$; $Vtc3^{TM1,mutant}$, $Vtc1/Vtc3_{K694A/K698A/R705A/R709A}/Vtc4$; $Vtc4^{TM1,mutant}$, $Vtc1/Vtc3/Vtc4_{K622A/R629A}$; $Vtc1^{\Delta N}$, $Vtc1_{\Delta 1\text{-}21}/Vtc3$ /Vtc4; $Vtc4^{TTM,mutant}$, $Vtc1/Vtc3/Vtc4_{K300A/R302A/K320A}$; $Vtc3^{TTM,mutant}$, $Vtc1/Vtc3_{K333A/K362A/K364A/Y380F}/Vtc4$. Source data are provided as a Source Data file. The experiments were repeated two times independently with similar results.

**Supplementary Table 1.**

Cryo-EM data collection and refinement statistics

| | PDB: 7YTJ<br>EMBD: EMD- 34090 |
|---|---|
| **Data collection and processing** | |
| Microscope | Krios |
| Voltage (kV) | 300 |
| Camera | Gatan K3 |
| Magnification | 81,000 |
| Pixel size at detector (Å/pixel) | 0.85 |
| Total electron exposure ($e^-$/Å$^2$) | 55 |
| Frames collected during exposure (no.) | 32 |
| Defocus range (μm) | -1.2~-2.2 |
| Automation software | EPU |
| Micrographs collected (no.) | 10,033 |
| Micrographs used (no.) | 9,861 |
| Total extract particles (no.) | 5,398,065 |
| | |
| **For each reconstruction** | |
| Refined particles (no.) | 734,934 |
| Final particles (no.) | 734,934 |
| Point group | C1 |
| Resolution (global, Å) | |
| FSC 0.5 (unmasked/masked) | 4.2/3.4 |
| FSC 0.143 (unmasked/masked) | 3.7/3.0 |
| Resolution range (local, Å) | 2.8-4.6 |
| Map sharpening B factor (Å$^2$) | 132.6 |
| Map sharpening methods | Half-maps correlation |
| | |
| **Model composition** | |
| Protein | 1,574 |
| Ligands | 7 |
| | |
| **Model refinement** | |
| Refinement package | PHENIX |
| - real or reciprocal space | Real Space |
| - resolution cutoff | 3.0 |
| Model-Map scores | |
| - CC | 0.79 |
| B factors (Å$^2$) | |
| Protein residues | 81.12 |
| Ligands/DNA/RNA | 93.25 |
| R.m.s. deviations from ideal values | |
| Bonds length (Å) | 0.003 |

| | |
|---|---|
| Bond angles (°) | 0.637 |
| **Validation** | |
| MolProbity score | 1.74 |
| CaBLAM outliers | 2.22 |
| Clashscore | 8.42 |
| Poor rotamers (%) | 1.27 |
| C-beta deviations | 0 |
| EMRinger score (if better than 4 Å resolution) | 2.51 |
| Ramachandran Plot | |
| Favored (%) | 96.72 |
| Outliers (%) | 0 |

**Supplementary Table 2.**

List of yeast strains used in this study

pr, promoter

| Stain name | Genotype | Background strain | Figure |
|---|---|---|---|
| Wt (BY4741) | *MATa his3Δ1 leu2Δ0 met15Δ0 ura3Δ0* | *MATa* | Fig.1 |
| *vtc1Δ* | *MATa his3Δ1 leu2Δ0 met15Δ0 ura3Δ0 vtc1::KanMX* | *BY4741* | Fig. 1 |
| *vtc2Δ* | *MATa his3Δ1 leu2Δ0 met15Δ0 ura3Δ0 vtc2::KanMX* | *BY4741* | Fig. 1 |
| *vtc3Δ* | *MATa his3Δ1 leu2Δ0 met15Δ0 ura3Δ0 vtc3::KanMX* | *BY4741* | Fig. 1 |
| *vtc4Δ* | *MATa his3Δ1 leu2Δ0 met15Δ0 ura3Δ0 vtc4::KanMX* | *BY4741* | Fig. 1 |
| *vtc1Δ vtc2Δ* | *MATa his3Δ1 leu2Δ0 met15Δ0 ura3Δ0 vtc1::KanMX vtc2::BleoR* | *vtc1Δ* | |
| *vtc2Δ vtc3Δ* | *MATa his3Δ1 leu2Δ0 met15Δ0 ura3Δ0 vtc2::BleoR vtc3::KanMX* | *vtc3Δ* | Fig.1 |
| *vtc2Δ vtc4Δ* | *MATa his3Δ1 leu2Δ0 met15Δ0 ura3Δ0 vtc2::BleoR vtc4::KanMX* | *vtc4Δ* | |
| *vtc1* | *vtc1Δ::KanMX vtc2Δ::BleoR vtc1* LEU2-ADHpr-N-2×Strep [pRS415-*vtc1*] | *vtc1Δ vtc2Δ* | Fig. 5b |
| $vtc1^{\Delta N}$ | *vtc1Δ::KanMX vtc2Δ::BleoR vtc1* LEU2-ADHpr-N-2×Strep [pRS415-$vtc1^{\Delta N}$] | *vtc1Δ vtc2Δ* | Fig. 5b |
| $vtc1^{TM1,mutant}$ | *vtc1Δ::KanMX vtc2Δ::BleoR vtc1* LEU2-ADHpr-N-2×Strep [pRS415-$vtc1^{TM1,mutant}$] | *vtc1Δ vtc2Δ* | Fig. 5b |
| *vtc3* | *vtc2Δ::BleoR vtc3Δ::KanMX vtc3* LEU2-ADHpr-N-Myc [pRS415-*vtc3*] | *vtc2Δ vtc3Δ* | Fig. 5b |
| $vtc3^{TM1,mutant}$ | *vtc2Δ::BleoR vtc3Δ::KanMX vtc3* LEU2-ADHpr-N-Myc [pRS415-$vtc3^{TM1,mutant}$] | *vtc2Δ vtc3Δ* | Fig. 5b |
| *vtc4* | *vtc2Δ::BleoR vtc4Δ::KanMX vtc4* LEU2-ADHpr-N-2×Strep [pRS415-*vtc4*] | *vtc2Δ vtc4Δ* | Fig. 5b |
| $vtc4^{TM1,mutant}$ | *vtc2Δ::BleoR vtc4Δ::KanMX vtc4* LEU2-ADHpr-N-2×Strep [pRS415-$vtc4^{TM1,mutant}$] | *vtc2Δ vtc4Δ* | Fig. 5b |

**Supplementary Table 3.**

List of primers used in this study

| Primer name | Sequence (5'-3') | Remark |
|---|---|---|
| **Primers for yeast construction** | | |
| *vtc2*Δ (FW) | CAACATAACGACACTTTTTTGACATGGTACAGA AGATTAAGTGAG | *vtc2* deletion primer |
| *vtc2*Δ (Rv) | CTCAGTAGATAGAGTACATATTCTACTTTTGCTC ACATGTTGGTCTC | *vtc2* deletion primer |
| KanMX (FW) | CCTCGACATCATCTGCCCAGATGCGAAGTTAAG TG | KanMX insert primer |
| KanMX (Rv) | GTTCTTTGTTGAATTTGTTGTCCACGGCTTCATC GTGTTG | KanMX insert primer |
| *vtc1*Δ (FW) | TCGCTATTTTCGGATAGAATGGCAAGAGCGATTA AG | upstream of *vtc1* gene |
| *vtc1*Δ (Rv) | CAGTTTGTGCGTAACCCACGCTTACGATATTG | downstream of *vtc1* gene |
| *vtc2*Δ (FW) | CACTGCGGTATAGCACTTCCAGAAAGATACTGA CTG | upstream of *vtc2* gene |
| *vtc2*Δ (Rv) | CAAAAACCGGAGAAAATGATGTTTATGTAAATG CATTG | downstream of *vtc2* gene |
| *vtc3*Δ (FW) | AAGGCAGAAACGTGGAAACATACACACACGCA CAC | upstream of *vtc3* gene |
| *vtc3*Δ (Rv) | CACCTGCTGAAAGCTGTTTAATATGATTTAATAC | downstream of *vtc3* gene |
| *vtc4*Δ (FW) | TGGTTTGTCTGCGTTTTGACGGAGAGCTACTGA CTTGTAG | upstream of *vtc4* gene |
| *vtc4*Δ (Rv) | GTGTGCGCCTGGTGAAGGTGTGCATTTCAGGCA AG | downstream of *vtc4* gene |
| *vtc1* (FW) | CCGCAGTTCGAAAAAGGATCCATGTCTTCAGCA CCATTATTAC | *vtc1* complement primer |
| *vtc1* (Rv) | GACGGTATCGATAAGCTT TTATAACTTAGTGTTAGCGTCATTG | *vtc1* complement primer |
| *vtc2* (FW) | CAAGGATGACGACGATAAGGGATCCATGCTGT TTGGAGTGAAG | *vtc2* complement primer |
| *vtc2* (Rv) | GACGGTATCGATAAGCTTCTAATCACTGCTTGG CCCCATTAACTTG | *vtc2* complement primer |
| *vtc3* (FW) | CGCTCTAGAACTAGTGGATCCATGGAGCAGAA ACTCATCTCTGAAG | *vtc3* complement primer |
| *vtc3* (Rv) | GACGGTATCGATAAGCTTTTATTCCCCAACCAA ATTGAAG | *vtc3* complement primer |
| *vtc4* (FW) | CCGCAGTTCGAAAAAGGATCCATGAAGTTTGG TGAGCACTTG | *vtc4* complement primer |
| *vtc4* (Rv) | GACGGTATCGATAAGCTTTTATTTAGCAACTAG GTTGCAGAAAAAG | *vtc4* complement primer |

| | | |
|---|---|---|
| *vtc1*$_{K24A/R31A}$ (FW) | GAGTTGAGCCAGCCGTGTTCTTTGCCAATGAGGCCACCTTTTTGTCG | *vtc1*$_{K24A/R31A}$ complement primer |
| *vtc1*$_{K24A/R31A}$ (Rv) | CGACAAAAAGGTGGCCTCATTGGCAAAGAACACGGCTGGCTCAACTC | *vtc1*$_{K24A/R31A}$ complement primer |
| *vtc1*$_{\Delta 1\text{-}21}$ (FW) | TGGAGCCACCCGCAGTTCGAAAAAATGGAGCCAAAAGTGTTCTTTG | *vtc*$_{\Delta 1\text{-}21}$ complement primer |
| *vtc1*$_{\Delta 1\text{-}21}$ (Rv) | CAAAGAACACTTTTGGCTCCATTTTTTCGAACTGCGGGTGGCTCCA | *vtc*$_{\Delta 1\text{-}21}$ complement primer |
| *vtc3*$_{K694A/K698A/R705A/R709A}$ (FW) | AATGCCGGTCCTGTCGCTGTTGAGGCAGCTGTTTGGCTCGCCAAT | K694A/K698A mutation site |
| *vtc3*$_{K694A/K698A/R705A/R709A}$ (Rv) | ATTGGCGAGCCAAACAGCTGCCTCAACAGCGACAGGACCGGCATT | K694A/K698A mutation site |
| *vtc3*$_{K694A/K698A/R705A/R709A}$ (FW) | AGTTTGGCTCGCCAATGAAGCCACATTCAATGCCTGGTTAAGTGTAACCAC | R705A/R709A mutation site |
| *vtc3*$_{K694A/K698A/R705A/R709A}$ (Rv) | GTGGTTACACTTAACCAGGCATTGAATGTGGCTTCATTGGCGAGCCAAACT | R705A/R709A mutation site |
| *vtc4*$_{K622A/R629A}$ (FW) | GTTCGTGTGGAACCAGCCGTTTACTTTGCCACTGAAGCCACCTACCTGTCTTGG | K622A/R629A mutation site |
| *vtc4*$_{K622A/R629A}$ (Rv) | CCAAGACAGGTAGGTGGCTTCAGTGGCAAAGTAAACGGCTGGTTCCACACGAAC | K622A/R629A mutation site |

**Primers for prokaryotic expression**

| | | |
|---|---|---|
| TTM$_{189\text{-}480}^{Vtc4}$ (FW) | GAAGGAGATATACATATGGGCAAGCAGCAAAATTTCGTGAGGCAG | TTM$_{189\text{-}480}^{vtc4}$ protein expression and purification |
| TTM$_{189\text{-}480}^{Vtc4}$ (Rv) | GTGATGGTGATGCTCGAGCTGGGGCAGCCAAAAGGGGATGGAG | TTM$_{189\text{-}480}^{vtc4}$ protein expression and purification |
| TTM$_{183\text{-}553}^{Vtc3}$ (FW) | AAGTTGATGCACATATGACCGTGTCCAAGTCCCTGGCTTCCACCAG | TTM$_{183\text{-}553}^{vtc3}$ protein expression and purification |
| TTM$_{183\text{-}553}^{Vtc3}$ (Rv) | CAGCCGGATCCTCGAGTTACAGGTCGGGCAGCCAAAAGGGCAG | TTM$_{183\text{-}553}^{vtc3}$ protein expression and purification |
| TTM$_{189\text{-}480}^{Vtc4,mutant}$ (FW) | GAAGGCCAGGTTTGCCCTGGCCGAGGCCCACGTGAATGAC | K300A/R302A mutant point |

| | | |
|---|---|---|
| $TTM_{189-480}^{Vtc4,mutant}$ (Rv) | GTCATTCACGTGGGCCTCGGCCAGGGCAAACCTGGCCTTC | K300A/R302A mutant point |
| $TTM_{189-480}^{Vtc4,mutant}$ (FW) | CCAGGTGTTTGCCGCCATGAGAAAGGAGGGCAAGAAG | K320A mutation site |
| $TTM_{189-480}^{Vtc4,mutant}$ (Rv) | CTTCTTGCCCTCCTTTCTCATGGCGGCAAACACCTGG | K320A mutation site |
| $TTM_{183-553}^{Vtc3,mutant}$ (FW) | ATCGGCAAGCTGCTGGACGCCCCCGATATCTTTCTGGAG | K333A mutation site |
| $TTM_{183-553}^{Vtc3,mutant}$ (Rv) | CTCCAGAAAGATATCGGGGGCGTCCAGCAGCTTGCCGAT | K333A mutation site |
| $TTM_{183-553}^{Vtc3,mutant}$ (FW) | GGAGATCAGACTGCAGATGGCCGCTGCCTTTATCAACAATTTCATC | K362/364A mutation site |
| $TTM_{183-553}^{Vtc3,mutant}$ (Rv) | GATGAAATTGTTGATAAAGGCAGCGGCCATCTGCAGTCTGATCTCC | K362/364A mutation site |
| $TTM_{183-553}^{Vtc3,mutant}$ (FW) | GACCCTAGCTACAAGAACTTCCTGATCAACCAGCTGAG | Y380F mutation site |
| $TTM_{183-553}^{Vtc3,mutant}$ (Rv) | CTCAGCTGGTTGATCAGGAAGTTCTTGTAGCTAGGGTC | Y380F mutation site |

**Primers for eukaryotic expression**

| | | |
|---|---|---|
| Vtc1 (FW) | ATTATCGATCCGGAGGTACCATGGCTTCCTCCGCCCCCCTG | VTC1 protein expression and purification |
| Vtc1 (Rv) | ATGGGTAGGCGCTCTCGAGCAGCTTGGTATTGGCATC | VTC1 protein expression and purification |
| Vtc3 (FW) | GATGACGACGATAAGGGATCCATGCTGTTTGGCATCAAGCTG | VTC3 protein expression and purification |
| Vtc3 (Rv) | CTGCTAGCAAGCTTCTCGAGTTACTCGCCCACCAGGTTAAAG | VTC3 protein expression and purification |
| Vtc4 (FW) | ATTATCGATCCGGAGGTACCATGGCTAAGTTCGGCGAGCAC | VTC4 protein expression and purification |
| Vtc4 (Rv) | GGCTCCAGGCGCTCTCGAGCTTGGCCACCAGGTTAC | VTC4 protein expression and purification |
| $Vtc4_{R264A/R266A/E426N}$ (FW) | GAGGCCCACGCCCTGGCGTGGTACGGCGGCATG | R264A/R266A mutation site |
| $Vtc4_{R264A/R266A/E426N}$ (Rv) | CATGCCGCCGTACCACGCCAGGGCGTGGGCCTC | R264A/R266A mutation site |

| | | |
|---|---|---|
| $Vtc4_{R264A/R266A/E426N}$ (FW) | TACGCCGTGCTGAACGTGAAGCTGCAGACCCAG | E426N mutation site |
| $Vtc4_{R264A/R266A/E426N}$ (Rv) | CTGGGTCTGCAGCTTCACGTTCAGCACGGCGTA | E426N mutation site |
| $Vtc3_{Y22F/K26A/K130A}$ (FW) | GATTCCTACATCGACTTCGAGAGGCTGGCTAAGCTGCTGAAGGAG | Y22F/K26A mutation site |
| $Vtc3_{Y22F/K26A/K130A}$ (Rv) | CTCCTTCAGCAGCTTAGCCAGCCTCTCGAAGTCGATGTAGGAATC | Y22F/K26A mutation site |
| $Vtc3_{Y22F/K26A/K130A}$ (FW) | TTCATCAAGATCGTGAAGGCTCACGATAAGCTGCAC | K130A mutation site |
| $Vtc3_{Y22F/K26A/K130A}$ (Rv) | GTGCAGCTTATCGTGAGCCTTCACGATCTTGATGAA | K130A mutation site |
| $Vtc4_{Y22F/K26A/K133A}$ (FW) | TACTACTACATCTCCTTCGACGACCTGGCTACAGAGCTGGAGGAC | Y22F/K26A mutation site |
| $Vtc4_{Y22F/K26A/K133A}$ (Rv) | GTCCTCCAGCTCTGTAGCCAGGTCGTCGAAGGAGATGTAGTAGTA | Y22F/K26A mutation site |
| $Vtc4_{Y22F/K26A/K133A}$ (FW) | CCAGAAGATCATCAAGGCTCACGATAAGAAGACAG | K133A mutation site |
| $Vtc4_{Y22F/K26A/K133A}$ (Rv) | CTGTCTTCTTATCGTGAGCCTTGATGATCTTCTGG | K133A mutation site |
| $Vtc3_{K126A/K129A/K133A}$ (FW) | GGCTTCATCGCTATCGTGGCTAAGCACGATGCTCTGCACCCCA | $Vtc3_{K126A/K129A/K133A}$ protein expression and purification |
| $Vtc3_{K126A/K129A/K133A}$ (Rv) | TGGGGTGCAGAGCATCGTGCTTAGCCACGATAGCGATGAAGCC | $Vtc3_{K126A/K129A/K133A}$ protein expression and purification |
| $Vtc4_{K129A/K132A/K136A}$ (FW) | GGCTTCCAGGCCATCATCGCCAAGCACGATGCCAAGACAG | $Vtc4_{K129A/K132A/K136A}$ protein expression and purification |
| $Vtc4_{K129A/K132A/K136A}$ (Rv) | GGCTTCCAGGCCATCATCGCCAAGCACGATGCCAAGACAG | $Vtc4_{K129A/K132A/K136A}$ protein expression and purification |
| $Vtc1_{K24A/R31A}$ (FW) | AGAGTGGAGCCTGCCGTGTTCTTCGCCAATGAGGCCACCTTTCTGTCCTG | $Vtc1_{K24A/R31A}$ protein expression and purification |
| $Vtc1_{K24A/R31A}$ (Rv) | CAGGACAGAAAGGTGGCCTCATTGGCGAAGAACACGGCAGGCTCCACTCT | $Vtc1_{K24A/R31A}$ protein |

| | | |
|---|---|---|
| | | expression and purification |
| Vtc1$_{\Delta 1\text{-}21}$ (FW) | ATTATCGATCCGGAGGTACCATGGAGCCTAAG GTGTTCTTCGCCAAT | Vtc1$_{\Delta 1\text{-}21}$ protein expression and purification |
| Vtc1$_{\Delta 1\text{-}21}$ (Rv) | ATGGGTAGGCGCTCTCGAGCAGCTTGGTATTGG CATC | Vtc1$_{\Delta 1\text{-}21}$ protein expression and purification |
| Vtc3$_{K694A/K698A/R705A/R709A}$ (FW) | GAACGCCGGACCTGTGGCTGTGGAGGCTGCTG TGTGGCTGGCTAATG | K694/R698A mutation site |
| Vtc3$_{K694A/K698A/R705A/R709A}$ (Rv) | GATGAAATTGTTGATAAAGGCAGCGGCCATCT GCAGTCTGATCTCC | K694/R698A mutation site |
| Vtc3$_{K694A/K698A/R705A/R709A}$ (FW) | TGTGGCTGGCTAATGAGGCTACCTTCAATGCTT GGCTGAGCGTGACAAC | R705/R709A mutation site |
| Vtc3$_{K694A/K698A/R705A/R709A}$ (Rv) | GTTGTCACGCTCAGCCAAGCATTGAAGGTAGCC TCATTAGCCAGCCACA | R705/R709A mutation site |
| Vtc4$_{K622A/R629A}$ (FW) | GTGAGAGTGGAGCCCGCCGTGTACTTTGCCACC GAGGCCACATACCTGAGCTGG | Vtc4$_{K622A/R629A}$ protein expression and purification |
| Vtc4$_{K622A/R629A}$ (Rv) | CCAGCTCAGGTATGTGGCCTCGGTGGCAAAGT ACACGGCGGGCTCCACTCTCAC | Vtc4$_{K622A/R629A}$ protein expression and purification |
| Vtc1$_{C105M}$ (FW) | GGACCTACACTGCTGATGTTTTTCCTGCTGGTG | VTC1 background Cys mutation |
| VTC1$_{C105M}$ (Rv) | CACCAGCAGGAAAAACATCAGCAGTGTAGGTC C | VTC1 background Cys mutation |
| Vtc3$_{C106M}$ (FW) | GAACACCCTGGAGGAGATGCTGGATGAGGCTC AGAG | VTC3 background Cys mutation |
| Vtc3$_{C106M}$ (Rv) | CTCTGAGCCTCATCCAGCATCTCCTCCAGGGTG TTC | VTC3 background Cys mutation |
| Vtc3$_{C751M}$ (FW) | CTTTCTGACCCTGTTCATGGGCGTGTGGGCTTA C | VTC3 background Cys mutation |
| Vtc3$_{C751M}$ (Rv) | GTAAGCCCACACGCCCATGAACAGGGTCAGAA AG | VTC3 background Cys mutation |

| | | |
|---|---|---|
| $Vtc4_{C63M}$ (FW) | AAGGTGTACACATTCATGAAGGTGAAGCACAGCGAGGTG | VTC4 background Cys mutation |
| $Vtc4_{C63M}$ (Rv) | CACCTCGCTGTGCTTCACCTTCATGAATGTGTACACCTT | VTC4 background Cys mutation |
| $Vtc4_{C418M}$ (FW) | GATGATAAGGATATCATGAGGTTCCCCTACGCCGTG | VTC4 background Cys mutation |
| $Vtc4_{C418M}$ (Rv) | CACGGCGTAGGGGAACCTCATGATATCCTTATCATC | VTC4 background Cys mutation |
| $Vtc4_{C614M}$ (FW) | CCGGCAAGACCATCATGGTGCCTGTGAGAGTGGAG | VTC4 background Cys mutation |
| $Vtc4_{C614M}$ (Rv) | CTCCACTCTCACAGGCACCATGATGGTCTTGCCGG | VTC4 background Cys mutation |
| $Vtc4_{C716M}$ (Rv) | GGCTCCAGGCGCTCTCGAGCTTGGCCACCAGGTTCATGAAAAAGC | VTC4 background Cys mutation |
| $Vtc4_{K415C}$ (FW) | CTTTTAAGCAGCTGGATGATTGTGATATCTGTAGGTTCCCCTACGCC | K415C mutation site |
| $Vtc4_{K415C}$ (Rv) | GGCGTAGGGGAACCTACAGATATCACAATCATCCAGCTGCTTAAAAG | K415C mutation site |
| $Vtc4_{R689C}$ (FW) | GGGTGGTGAACATCAGGCTGAAGTGTGCCGTGGATTACGAGGATAAGATC | R689C mutation site |
| $Vtc4_{R689C}$ (Rv) | GATCTTATCCTCGTAATCCACGGCACACTTCAGCCTGATGTTCACCACCC | R689C mutation site |

# Supplementary References


1 Aschar-Sobbi, R. et al. High sensitivity, quantitative measurements of polyphosphate using a new DAPI-Based approach. *Journal of Fluorescence* **18**, 859-866 (2008).

2 Hothorn, M. et al. Catalytic Core of a Membrane-Associated Eukaryotic Polyphosphate Polymerase. *Science* **324**, 513-516 (2009).

3 Wild, R. et al. Control of eukaryotic phosphate homeostasis by inositol polyphosphate sensor domains. *Science* **352**, 986-990 (2016).

4 Smart, O. S., Neduvelil, J. G., Wang, X., Wallace, B. A. & Sansom, M. S. P. HOLE: A program for the analysis of the pore dimensions of ion channel structural models. *Journal of Molecular Graphics & Modelling* **14**, 354-360 (1996).

5 Schwieters, C. D., Bermejo, G. A. & Clore, G. M. Xplor-NIH for molecular structure determination from NMR and other data sources. *Protein Science* **27**, 26-40 (2018).
